# A Virus-Agnostic Cellular Immunomodulatory Platform for Chronic Respiratory Disease: Restoring Immune Competence and Mitigating Exacerbations in the Elderly

**DOI:** 10.3390/vaccines14060475

**Published:** 2026-05-27

**Authors:** Michael Har-Noy

**Affiliations:** Immunovative Therapies, Ltd., Jerusalem 9610203, Israel; harnoy@immunovative.com

**Keywords:** Allopriming, immunosenescence, chronic obstructive pulmonary disease (COPD), asthma, viral-triggered exacerbations, interferon gap, pathogen-agnostic vaccine, mucosal immunity, lung-resident memory T cells (Trm), trained immunity

## Abstract

Chronic respiratory diseases (CRDs) represent a significant global mortality burden, largely driven by viral-triggered exacerbations. In the elderly, susceptibility to viral pathogens is critically linked to the “interferon gap”—a kinetic delay in innate antiviral signaling resulting from immunosenescence and Th2-skewed inflammaging. While traditional vaccines provide pathogen-specific protection, their efficacy is often compromised by age-related immune hyporesponsiveness and antigenic drift. This perspective paper proposes a dual-phase, virus-agnostic immunomodulatory platform designed to restore mucosal immune competence and provide a rapid-response intervention for incipient exacerbations. Rather than acting as a pathogen-specific vaccine, the platform serves as a comprehensive host immune-rejuvenation engine and cellular adjuvant platform. The platform consists of two integrated stages: Allopriming and Alloantigen Inhalation Recall (AIR). Allopriming utilizes AlloStim^®^ (activated, allogeneic Th1 cells) to leverage the evolutionarily conserved allo-rejection response, establishing a lung mucosal reservoir of allo-specific Th1 tissue-resident memory cells (Trm). Building on previously published Phase I/II data showing that Allopriming reverses biomarkers of immunosenescence and sustains durable heterologous antiviral responsiveness, the AIR strategy is introduced as a patient-administered rescue mechanism for frail CRD patients. AIR is designed to activate pre-positioned Trm cells at the earliest onset of symptoms, inducing a high-magnitude IFN-γ surge in the lung mucosa. By bridging the senescent “interferon gap” with the rapid effector kinetics of Trm activation, this approach represents a novel paradigm toward reconstituting youthful-like antiviral mucosal immunity to both enhance vaccine efficacy in the elderly and protect against both seasonal pathogens and emerging viral triggers (“Disease X”) of CRD. Future randomized studies in long-term care settings are planned to evaluate clinical outcomes in high-risk populations.

## 1. Introduction

Chronic respiratory diseases (CRDs), most notably Chronic Obstructive Pulmonary Disease (COPD) and asthma, represent an escalating global health crisis. COPD currently ranks as the third leading cause of death worldwide, affecting more than 400 million individuals [1], while asthma is estimated to affect over 260 million [2]. Asthma remains the second leading cause of mortality among chronic respiratory illnesses, contributing to over 400,000 deaths annually [3]. The clinical course of these diseases is punctuated by acute exacerbations—sudden intensifications of respiratory symptoms that drive accelerated lung function decline, increased hospitalization rates, and significant mortality [4]. Respiratory viruses are the primary catalysts for these events, detected in up to 80% of asthma and 40–60% of COPD exacerbations [5]. In the elderly population, these flare-ups are predominantly associated with a triad of seasonal viral pathogens: human rhinovirus (HRV), influenza, and respiratory syncytial virus (RSV) [6,7].

Immunization remains the cornerstone of preventive medicine for these vulnerable cohorts [8]. Vaccinations against influenza, pneumococcus, and, more recently, SARS-CoV-2 and RSV, are vital to mitigating life-threatening CRD complications [9]. Reflecting this urgency, the 2025 GOLD Strategy Report now explicitly mandates RSV vaccination for patients over 60 and the adoption of 21-valent pneumococcal vaccines (PCV21) to broaden protection against secondary bacterial sequelae [10]. However, despite rigorous advocacy by international health agencies, the global burden of CRDs remains substantial. The efficacy of current immunization strategies is frequently compromised by the unique physiological and immunological challenges of the aging lung, where “vaccine fatigue”, antigenic drift, and age-related hyporesponsiveness culminate in clinical failure [11,12].

The heightened susceptibility of the elderly is driven by the intertwined processes of immunosenescence and inflammaging [13,14]. Immunosenescence manifests as a fundamental dysregulation of the cellular immune system, specifically a progressive functional shift toward a Type 2-dominant (Th2) cytokine profile [15,16]. This Th2-dominance not only suppresses the maturation of high-affinity antibody responses required for traditional vaccine efficacy but also impairs the production of critical antiviral mediators, including Type I (IFN-α/β) and Type III (IFN-γ) interferons [17,18].

**Figure 1:**
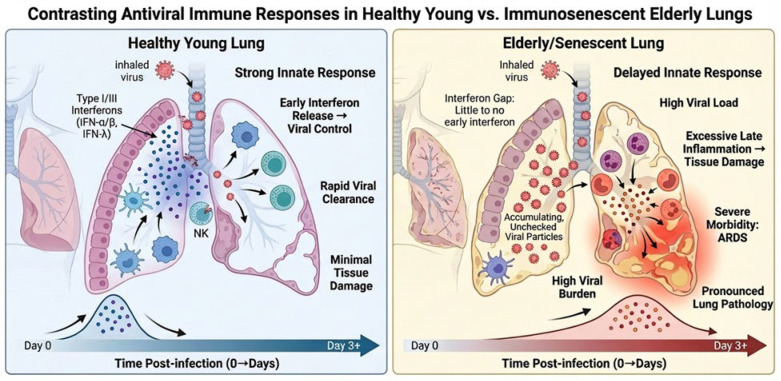
Antiviral Immune Kinetics in the Healthy Young vs. Immunosenescent Elderly Lung. In the healthy young lung (left panel), inhaled viral challenge triggers an immediate and robust release of Type I and III interferons (IFN-α/β, IFN-ϒ). This early innate peak (Day 0–1) effectively controls viral replication, leading to rapid clearance and minimal structural tissue damage. Conversely, the elderly/senescent lung (right panel) is characterized by a “Th2-skewed” microenvironment that creates a critical interferon gap. This kinetic delay in innate signaling allows for the unchecked accumulation of viral particles (High Viral Load). The failure of early viral control necessitates a compensatory, yet maladaptive, late-phase inflammatory surge (Day 3+). This excessive late inflammation does not resolve the infection but instead drives pronounced lung pathology, tissue damage, and severe clinical morbidity, including acute respiratory distress syndrome (ARDS) and mortality.

In the aged and diseased lung, a microenvironment characterized by elevated Th2 cytokines, IL-4 and IL-13, actively suppresses the capacity of airway epithelial cells to initiate robust interferon signaling. This creates a critical “interferon gap”, a kinetic delay in the innate antiviral response that allows viral replication to bypass early checkpoints. While the healthy young lung achieves rapid viral clearance through early interferon release, the senescent lung exhibits an unchecked increase in viral burden. This gap facilitates a transition from inadequate early defense to an excessive, maladaptive late-phase inflammatory response, resulting in pronounced lung pathology and severe morbidity (see Figure 1) [19,20].

Efforts to bridge the interferon gap via exogenous supplementation (e.g., inhaled IFN-β) have faced significant hurdles, primarily because the narrow therapeutic window for administration is rarely met in real-world outpatient settings [21,22]. Furthermore, passive supplementation fails to remodel the underlying Th2-dominant environment of the senescent lung. To address this, a strategy capable of counteracting both known seasonal threats and emerging ‘Disease X’ pathogens is required [23]. Such a platform must move beyond reactive supplementation toward the functional rejuvenation of youthful cellular immune kinetics.

To clarify the distinct therapeutic mechanisms of the Allopriming/AIR platform, Table 1 provides a direct comparative overview against standard seasonal vaccines and exogenous interferon supplementation. Crucially, the platform’s utility extends beyond antiviral defense to mitigate secondary bacterial sequelae caused by encapsulated pathogens such as *Streptococcus pneumoniae*. Although anti-bacterial immunity is predominantly antibody-mediated, the platform’s induction of a robust Th1 profile and localized IFN-γ secretion acts as an upstream multiplier of humoral function. IFN-γ serves as a key signaling cytokine that drives B-cell differentiation, affinity maturation, and immunoglobulin (Ig) class-switching toward highly efficient opsonizing IgG antibodies (such as IgG1 and IgG3 in humans). These antibodies work synergistically with IFN-γ-activated alveolar macrophages to accelerate bacterial clearance.

Clinical validation of this cross-talk is supported by our Phase I/II trial data, which demonstrated that Allopriming significantly enhanced neutralizing antibody titers of vaccinated elder adults. By functioning as a systemic and mucosal cellular adjuvant engine, this host-remodeling strategy reverses immunosenescence to substantially augment both the functional antibody and cellular responses to critical bacterial immunizations, such as the 21-valent pneumococcal conjugate vaccine (PCV21) mandated by the GOLD guidelines.

To address the systemic and mucosal limitations of current approaches, this perspective paper proposes a novel two-phase immunomodulatory platform: adjuvanted immunization with alloantigens (Allopriming) using living, activated allogeneic Th1 cells (AlloStim^®^) to create Th1 allo-specific immunity, coupled with Alloantigen Inhalation Recall (AIR). This combination strategy leverages the potency of the allogeneic response to remodel the senescent immune landscape by shifting the systemic Th1/Th2 balance and populating the lung mucosa with a reservoir of allo-specific Th1 tissue-resident memory T cells (Trm). These Trm act as ‘innate-like’ sentinels that can release IFN-γ upon a viral encounter through bystander activation, which can bridge the interferon gap. The AIR strategy provides a patient-initiated, targeted ‘rescue’ surge of IFN-γ via inhaled alloantigens to specifically activate resident allo-specific Trm at the earliest onset of symptoms.

Crucially, while Type I and Type III interferons constitute the classical innate response in a youthful lung, the Allopriming/AIR platform is designed to functionally replace the late or missing innate signal with Trm-derived IFN-γ [24]. Accordingly, by combining continuous mucosal surveillance with high-magnitude recall, this proposed dual-action platform aims to abort viral-triggered CRD exacerbations before they escalate into clinical crises.

## 2. Allopriming: AlloStim^®^ as a Cellular Adjuvant for Th1 Immunity

The experimental therapeutic, AlloStim^®^ is conceptually derived from the Graft-versus-Tumor (GvT) and Graft-versus-Host (GvH) mechanisms observed in allogeneic hematopoietic stem cell transplantation. The objective is to harness the potency of these reactions to create a Host-versus-Tumor (HvT) effect, driven by a controlled, clinically insignificant, Host-versus-Graft (HvG) rejection [25].

AlloStim^®^ (Immunovative Therapies, Ltd., Jerusalem, Israel) consists of living, mismatched, activated Th1 memory cells (CD4+, CD45RO+, CD40L^hi^, CD62L^lo^, CD25+) differentiated and expanded ex vivo from naïve healthy donor CD4+ T cells. In the final formulation, AlloStim^®^ cells are conjugated with anti-CD3/anti-CD28-coated microbeads in a 1:1 ratio to maintain constitutive activation, characterized by high-density CD40L expression and the secretion of inflammatory cytokines, including IFN-γ, TNF-α, and GM-CSF.

Distinct from conventional vaccines that primarily utilize adjuvants to polarize toward a Th2-biased humoral response, AlloStim^®^ functions as a self-contained, adjuvanted immunomodulatory therapy designed to immunize the host against donor alloantigens and polarize the resulting response toward a Th1-dominant phenotype. The intradermal (ID) injection of these activated allogeneic Th1 cells creates a localized pro-inflammatory milieu—a “danger zone”—that serves as a potent adjuvant for host allo-specific Th1 immunity. This iterative process of multiple ID injections, termed ‘Allopriming’, acts as an “immunological engine” designed to remodel the systemic immune landscape, shifting it from a Th2-biased senescent profile toward a Th1-biased state of youthful competence [26].

### 2.1. The Immunological Engine: Origin and Protocol Standardization

The experimental therapeutic, AlloStim^®^, originated as an advanced cellular immunomodulator within oncology frameworks, designed to elicit host-versus-tumor (HvT) responses via controlled allogeneic rejection cascades. When delivered intravenously or intratumorally in late-stage, heavily pre-treated cancer patients, intradermal administration has historically been associated with transient Adverse Events (AEs), including fever, chills and local site reactions. Intravenous infusion of primed cancer patients results in AEs related to inflammation and swelling of tumor lesions, such as non-specific pain, fatigue, and other immune-related adverse events (irAE). Inflammation of tumors also causes mass effect-related AEs, depending on the tumor size and location, such as bowel obstruction, hydronephrosis, superior vena cava (SVC) syndrome, bile duct obstruction and pulmonary embolism. However, when transitioned to geriatric respiratory medicine, the intradermal priming protocol has demonstrated a high safety profile.

The Allopriming regimen consists of a 14-day cycle comprising five sequential intradermal (ID) injections administered on Days 0, 3, 7, 10, and 14. Each standardized 0.5 mL dose contains 5 × 10^6^ viable, mismatched, ex vivo expanded and differentiated donor Th1 memory cells (CD4+, CD45RO+, CD40L^hi^, CD62L^Lo+^, CD25+, IFN-γ+, IL-4^−^, TNF-α+, GM-CSF+) conjugated to anti-CD3/anti-CD28-coated microbeads in a 1:1 formulation ratio. Each batch is quality tested for function with supernatants from the activated cells analyzed for ability to cause maturation of a dendritic cell (DC) cell line (THP-1) to upregulate CD86 and produce IL-12 in ex vivo cultures. Manufacturing consistency is regulated via strict Current Good Manufacturing Practice (cGMP) standards and mandatory quality control release criteria, including USP<71> sterility and USP<85> endotoxin testing. The iterative ID delivery creates a localized pro-inflammatory “danger zone” that licenses host DC into a pro-inflammatory DC1 phenotype, initiating a robust systemic-to-mucosal flux of allo-specific Th1 cells into the lung parenchyma.

The injection of living, activated, mismatched allogeneic cells into the dermis creates an “Immunological Engine” for continuous enhancement of cellular immune function over time. Upon injection, the anti-CD3/anti-CD28 bead-activated allogeneic Th1 cells establish a sustained pro-inflammatory ‘danger zone’ microenvironment at the injection site. The activated Th1 cells produce IFN-γ and TNF-α after injection, which establishes a Type 1-polarized interface. Since AlloStim^®^ is completely mismatched to the host, the cells undergo rejection by host NK cells, triggering the release of internal chaperoned alloantigens and danger-associated molecules (DAMP) into the pre-conditioned inflammatory microenvironment. Simultaneously, released GM-CSF actively recruits host Langerhans cells (LCs) and dermal DCs to the injection site, facilitating rapid alloantigen uptake and processing.

**Figure 2:**
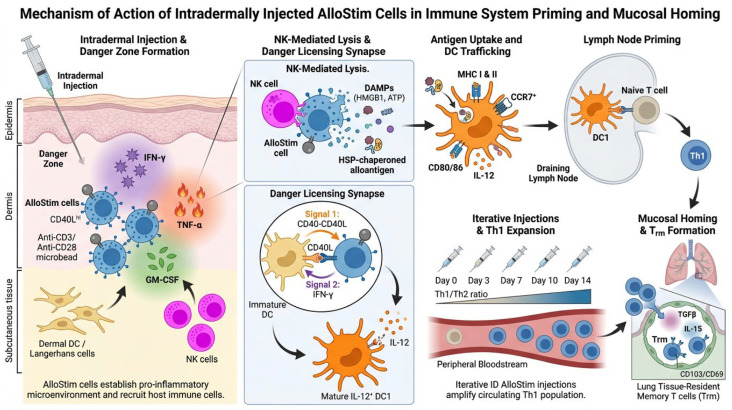
Mechanism of Action of Allopriming: The Allopriming phase establishes a systemic-to-mucosal flux of allo-specific Th1 immunity through a multi-step process: Intradermal “Danger Zone” Formation (left): Injected AlloStim^®^ cells (activated allogeneic Th1 cells) establish a pro-inflammatory microenvironment via the secretion of IFN-γ, TNF-α, and GM-CSF. This milieu recruits host Langerhans and dermal dendritic cells (DCs). Danger Licensing and Antigen Uptake (center): Host NK cells initiate Host-versus-Graft (HvG) rejection, leading to the immunogenic cell death of the allogeneic cells and the release of HSP-chaperoned alloantigens and DAMPs. Crucially, the high-density CD40L expression on AlloStim^®^ ensures host DCs are “licensed” via Signal 1 (CD40-CD40L) and Signal 2 (IFN-γ) prior to antigen processing. Lymph Node. Priming and Mucosal Sequestration (Right): Licensed DC1s migrate to draining lymph nodes to drive the clonal expansion of an allo-specific Th1 repertoire. Iterative injections (Days 0–14) amplify this population, which eventually utilizes mucosal homing signatures to extravasate into the lung parenchyma. Trm Differentiation (Bottom): Under the local influence of TGF-β and IL-15, these effectors differentiate into persistent, CD103+/CD69+ tissue-resident memory T cells (Trm), creating a pre-positioned cellular substrate for rapid, virus-agnostic defense.

Within this inflammatory milieu, the convergence of DAMP-mediated signaling and high-density CD40L expression ensures the “licensing” of host DCs into a pro-inflammatory DC1 phenotype. The iterative intradermal injections subsequently amplify the circulating titer of allo-specific Th1 cells, which eventually extravasate to the lung mucosa to establish a persistent anti-alloantigen-specific Trm reservoir. This systemic-to-mucosal flux, from initial intradermal ‘danger zone’ formation to the sequestration of lung-resident Trm, is detailed in Figure 2.

### 2.2. Host-Versus-Graft (HvG) Rejection and DC1 Licensing

The priming sequence culminates in a potent HvG response, a mechanism historically characterized by the Normal Lymphocyte Transfer (NLT) test [27]. Notably, this allo-rejection remains functional even in aged or immunocompromised hosts who are otherwise anergic to standard vaccines [28]. This suggests that the HvG response is an evolutionarily prioritized defense that can bypass the restricted T-cell receptor (TCR) diversity typical of the aged host [29].

The pre-activated state of AlloStim^®^, characterized by high-density surface expression of CD40L, ensures that host DCs are ‘licensed’ via direct CD40-CD40L interaction prior to cell clearance. This temporal coordination ensures host DC licensing is coupled to the subsequent allo-antigen release during the HvG response. During the subsequent rejection phase, host NK cells and macrophages induce “immunogenic cell death” (ICD) of the AlloStim^®^ cells [30], triggering a substantial release of endogenous danger signals (e.g., HMGB1, ATP) and Heat Shock Proteins (HSPs) [31]. These released HSPs act as molecular chaperones, binding donor alloantigens from the rejected cellular adjuvant for receptor-mediated endocytosis by host DCs [32]. Once internalized, these donor alloantigens are processed for canonical presentation on MHC Class II and cross-presentation on MHC Class I molecules. These newly licensed, antigen-loaded DCs then upregulate CCR7, express IL-12 and migrate to the draining lymph nodes. where they orchestrate the clonal expansion and differentiation of a robust, allo-specific Th1 and CD8+ T-cell repertoire.

### 2.3. Systemic Remodeling and Mucosal Homing

The induction phase of Allopriming modulates the systemic Th2 bias of the aged host by generating a robust systemic repertoire of allo-specific Th1 and CD8+ effector cells. During lymph node priming, a critical subset of these T cells is imprinted with mucosal homing signatures (e.g., integrin α4β1/VLA-4, CCR10 and CXCR3 [33,34] allowing them to egress into circulation and extravasate into the lung parenchyma. Under the influence of localized TGF-β and IL-15, these effectors differentiate into persistent tissue-resident memory T cells (Trm) (CD103+/CD69+) [35].

This pre-positioned cellular substrate enables a rapid, virus-agnostic defense via bystander activation triggered by innate DC sensing. This mechanism is analogous to the “Allogeneic Effect” [36], providing immediate protection by bypassing the requirement for direct viral antigen recognition. The resulting Trm-derived IFN-γ wave creates a localized antiviral state that concurrently facilitates the development of pathogen-specific adaptive immunity. In this polarized microenvironment, viral antigens released via viral- or NK-induced ICD are captured by resident DCs that have transitioned to a pro-inflammatory DC1 phenotype. These licensed DCs then orchestrate the priming of naïve T cells within localized lymphoid structures.

### 2.4. Self-Amplifying Mucosal Immunity Remodeling

The Type I-polarized mucosal microenvironment activates host NK cells and catalyzes the targeted lysis of virally infected host cells. This process provides the requisite conditions for in situ vaccination [37,38]. Unlike silent apoptosis, the immune-mediated destruction of infected cells via granzyme/perforin release constitutes a form of “immunological cell death” (ICD) [39,40], ensuring the co-release of viral neoantigens and endogenous danger-associated molecular patterns (DAMPs), such as HMGB1 and calreticulin.

In this inflammatory milieu, resident respiratory DCs are “licensed” to mature into a pro-inflammatory DC1 phenotype [30,41]. These DCs efficiently capture and cross-present viral antigens to naïve T cells, likely within inducible Bronchus-Associated Lymphoid Tissue (iBALT) or draining lymph nodes [42]. These iBALT structures serve as “intrapulmonary” priming hubs by bypassing the requirement for systemic lymph node trafficking. iBALT-mediated priming enables the rapid, local expansion of pathogen-specific cellular immunity directly at the site of viral entry.

**Figure 3:**
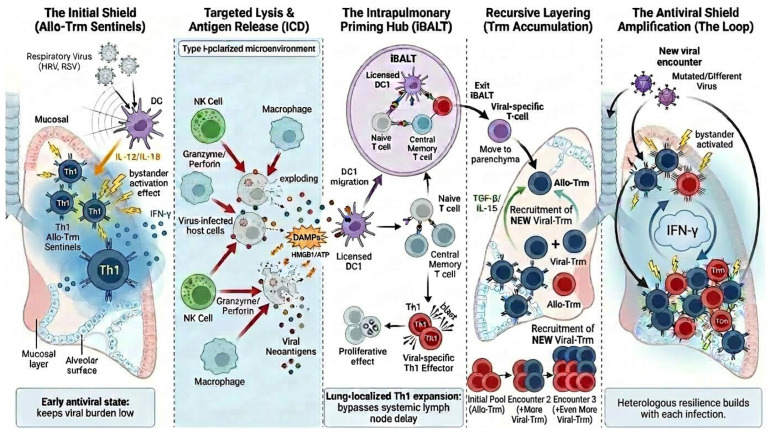
Self-Amplifying Mucosal Remodeling.

In this manner, the platform leverages the invading virus as a source of endogenous antigens to overlay a virus-specific memory layer atop the existing virus-agnostic allo-memory reservoir (see Figure 3). The established allo-specific Trm population acts as a functional ‘biological scaffold,’ ensuring that newly differentiated viral-specific Th1 effectors are recruited to the lung parenchyma to join the resident allo-specific Trm pool. This creates a self-amplifying positive feedback loop: each subsequent viral encounter not only expands the total Trm density but also broadens the localized T-cell repertoire. Consequently, the susceptible senescent lung is functionally remodeled over time, transforming from a state of vulnerability to one of progressively augmented, heterologous resilience.

The Allopriming platform leverages a recursive immunological cycle to transform the vulnerable, senescent lung into a site of robust antiviral defense: The Initial Shield: Pre-positioned allo-specific Th1 Trm sentinels undergo non-specific bystander activation (via IL-12/IL-18) upon viral encounter, releasing an immediate wave of IFN-γ to keep viral burden low. Targeted Lysis and ICD: In the polarized microenvironment, NK cells and macrophages induce Immunogenic Cell Death (ICD) of infected host cells, releasing viral neoantigens and DAMPs (e.g., HMGB1, ATP). The iBALT Hub: Licensed DC1s capture these antigens and migrate to inducible Bronchus-Associated Lymphoid Tissue (iBALT), bypassing systemic lymph node delays to drive local expansion of viral-specific Th1 effectors. Recursive Layering: Newly differentiated viral-specific Trm join the existing allo-specific “scaffold,” cumulatively increasing the total Trm density and repertoire diversity with each infection encounter. The Loop: Subsequent encounters trigger a high-magnitude, accelerated IFN-γ response upon each environmental virus encounter from the expanded, diversified Trm pool, establishing progressively augmented heterologous resilience.

### 2.5. Clinical Foundation and Strategic Evolution

In contrast to the pathogen-specific focus of traditional vaccines, the Allopriming platform is designed to enhance protection through subsequent environmental exposures. This recursive competence was demonstrated in a Phase I/II clinical trial involving healthy adults over 65 years old [43]. Allopriming was well-tolerated with a favorable safety profile, confirming that the high-magnitude Th1 stimulus remains clinically manageable. Specifically, intradermal Allopriming was occasionally associated primarily with transient, localized Injection Site Reactions (ISRs)—including erythema, induration, and mild tenderness—which resolved spontaneously within 24–48 h without medical intervention. Crucially, longitudinal safety monitoring out to Day 336 revealed zero Serious Adverse Events (SAEs), no instances of systemic cytokine release syndrome (CRS), and no clinical or laboratory evidence of induced auto-reactive or autoimmune complications.

## 3. Alloantigen Inhalation Recall (AIR)

**Figure 4:**
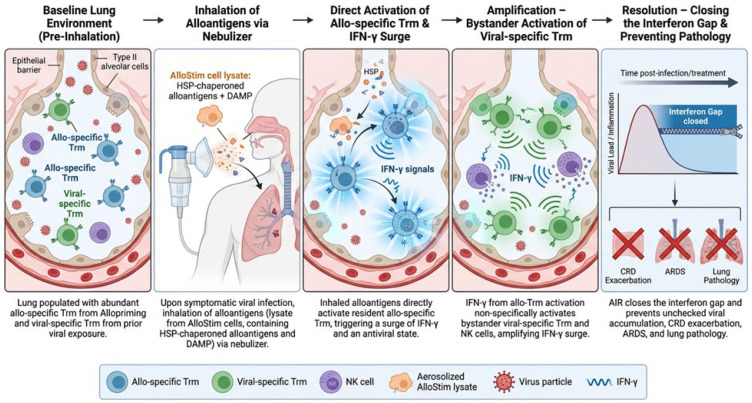
Mechanism of Alloantigen Inhalation Recall (AIR) and Closing the Interferon Gap.

The Alloantigen Inhalation Recall (AIR) phase utilizes the inhalation of specific alloantigens to trigger a localized, high-magnitude immune response. AIR consists of a lyophilized powder of lysed AlloStim^®^ cells, reconstituted and administered via a nebulizer at the earliest onset of respiratory symptoms. This recall mechanism is designed to provide a rapid, virus-agnostic “antiviral shield” by promoting a substantial localized surge of IFN-γ through the direct reactivation of lung-resident, allo-specific Trm memory cells. This induced state mimics the kinetics of trained immunity, supplementing the impaired innate responses of the elderly and diseased lung (see Figure 4).

The AIR phase provides a rapid, high-magnitude surrogate signal to functionally replace the missing innate antiviral response in the senescent lung. Baseline: Following Allopriming and prior environmental challenges, the lung mucosa is pre-populated with a scaffold of allo-specific Trm (blue) and viral-specific Trm (green). Inhalation: Upon early symptom onset, nebulized AlloStim cell lysate (containing HSP-chaperoned alloantigens and DAMPs) is delivered to the airways. Direct Activation: Inhaled alloantigens engage resident allo-specific Trm, triggering a “Day Zero” surge of IFN-γ and establishing an immediate antiviral state. Bystander Amplification: Allo-Trm-derived IFN-γ facilitates the non-specific bystander activation of resident viral-specific Trm and NK cells (purple), further amplifying the localized IFN-γ peak. Resolution: By closing the “interferon gap” at the earliest stage of infection, the AIR-induced shield prevents unchecked viral replication and the maladaptive inflammatory cascade, thereby aborting clinical CRD exacerbations, ARDS, and permanent lung pathology.

### 3.1. The IFN-γ Surge and Bystander Amplification

By activating lung-resident allo-specific Trm cells via inhaled alloantigen, the AIR phase triggers an allo-specific recall that results in the immediate local release of IFN-γ. This primary signal leverages the high frequency and pre-positioned nature of the allo-specific memory pool to induce an innate-like, virus-agnostic antiviral state. Extracellular, inert viral particles are neutralized by pathogen-specific antibodies upon entry into the respiratory tract. The AIR-mediated release of IFN-γ does not prevent initial viral adherence or cellular entry; rather, it functions immediately after the intracellular replication cycle has been initiated. By triggering a localized, high-magnitude wave of IFN-γ via bystander activation of pre-positioned mucosal Trm cells at the very first sign of cellular infection, the platform acts as an acute replication circuit breaker. This rapid intracellular containment limits overall viral shedding and load, keeping the infection below the threshold required to trigger severe lung tissue pathology.

Crucially, this cytokine release also facilitates the bystander activation of natural killer (NK) cells and existing viral-specific Th1 cells within the mucosal microenvironment [44]. This localized cytokine surge effectively “pre-conditions” the lung, suppressing viral replication before it reaches peak titers and bridges the transition to an adaptive, viral-specific response. In patients with CRD, this rapid intervention is intended to abort the viral replication cycle that otherwise drives the high-magnitude inflammatory cascades responsible for acute exacerbations, thereby preventing the transition to severe clinical morbidity.

### 3.2. The Mucosal Relay: Biphasic IFN-γ Introduction

Upon AIR deposition, the aerosolized droplets merge with the epithelial lining fluid, liberating HSP-chaperoned alloantigens for capture by resident DCs and direct contact with Trm cells.

#### 3.2.1. Low-Threshold Trm Reactivation

Because these Trm cells are pre-positioned in the mucosal niche, they bypass the stringent requirements of naïve T-cell activation. They require only a TCR-mediated signal (Signal 1) to initiate effector function, eliminating the need for the B7-CD28 costimulatory “licensing” typically required in systemic lymph nodes [45,46], thus facilitating near-instantaneous IFN-γ synthesis.

#### 3.2.2. Bystander Amplification

This initial IFN-γ wave is proposed to catalyze a secondary, non-specific cascade. The resulting cytokine-rich microenvironment triggers neighboring NK cells and resident bystander Th1 cells to release a secondary wave of IFN-γ and IL-12. This bifurcated response aims to establish a virus-agnostic “antiviral shield” that suppresses replication with kinetics approximating a youthful innate response [44].

#### 3.2.3. Self-Amplifying Defense

The high-magnitude Type 1 environment provides the necessary chemotactic cues (e.g., CXCL13, CCL19) for the assembly of inducible Bronchus-Associated Lymphoid Tissue (iBALT). Here, host DCs capture viral fragments released during ICD and, under the influence of the established IFN-γ/IL-12 milieu, mature into a pro-inflammatory DC1 phenotype. This effectively converts the site of infection into a functional tertiary lymphoid structure for de novo priming and memory maintenance, strengthening long-term mucosal resilience against both homologous and heterologous viral encounters.

### 3.3. A Transformative Shift in CRD Management

The synergy of Allopriming and AIR represents a potential paradigm shift in CRD management. By functionally remodeling the systemic immune architecture and providing a localized, rapid-response trigger, this platform can bridge the “interferon gap.” Unlike strain-specific vaccines that are vulnerable to antigenic drift, this strategy could establish a pre-emptive, self-amplifying antiviral state that is both virus-agnostic and durable. This approach offers a robust framework for protecting vulnerable populations against both known seasonal threats and emerging “Disease X” pathogens.

## 4. Technical Specifications and Clinical Delivery

### 4.1. Formulation and Stability: The Lyophilized-to-Liquid Bridge

The platform utilizes a lyophilized-to-liquid bridge to ensure pharmaceutical stability and clinical ease of use. While the Allopriming induction phase requires viable cells for systemic licensing, the AIR rescue phase utilizes a fixed-antigen lysate. This process is optimized to release biologically active Hsp70/90-alloantigen complexes and endogenous DAMPs using cryoprotectants to maintain the functional folding of chaperoned alloantigens and mitigate protein aggregation [47]. This stable formulation allows for at-home storage, ensuring that the patient can reconstitute the vial with sterile saline at the earliest onset of symptoms to provide a high-potency protective “trigger” when the therapeutic window is narrowest.

### 4.2. Nebulization Mechanics and Airway Tolerability

For elderly patients with pre-existing CRDs, vibrating mesh nebulizers offer a superior delivery vehicle compared to Dry Powder Inhalers (DPIs), which require a forceful inspiratory effort often unattainable during acute bronchospasm [48]. The AIR rescue phase utilizes a single-dose fixed-antigen lysate (3 × 10^7^ AlloStim^®^ cell equivalents) tested for preservation of alloantigen chaperone proteins (e.g., HSP70, HSP96, gp96) and DAMP (e.g., HMGB1, ATP) after lyophilization and formulated with cryoprotectants (e.g., trehalose) and reconstituted in 3 mL of sterile 0.9% isotonic saline. Reconstitution with 0.9% saline ensures an isotonic, pH-balanced aerosol, minimizing the paradoxical bronchoconstriction associated with high-mass median diameter droplets.

Modern mesh devices produce a Mass Median Aerodynamic Diameter (MMAD) of 1–5 µm, optimized for deep lung deposition at the bronchioloalveolar junctions—the primary anatomical niche for pre-positioned Trm cells [49]. Crucially, because this aerosolized route represents a novel delivery mechanism for a cellular lysate, it must be approached with high clinical caution in patients with hyper-reactive airways. The initiation of any clinical evaluation strictly mandates a preliminary Phase I human safety and tolerability dose-escalation trial to establish baseline airway safety prior to assessing larger clinical efficacy endpoints.

## 5. Discussion and Future Perspectives

The Allopriming platform represents a fundamental paradigm shift in geriatric respiratory medicine, moving from the reactive supplementation of missing mediators to the proactive remodeling of the host’s cellular architecture.

### 5.1. Addressing “Disease X” and the 100-Day Mission

Global health authorities have established the “100-Day Mission” to deploy pathogen-specific vaccines within 100 days of identifying a “Disease X” threat [50]. However, the COVID-19 pandemic demonstrated that even a 100-day window represents a period of catastrophic risk for the elderly and frail [51]. The Allopriming/AIR platform provides a concrete, actionable implementation pathway to bypass this vulnerability. Rather than waiting for a novel pathogen to be isolated and sequenced, high-risk populations would receive routine, one-time Allopriming during ‘blue sky’ intervals in order to establish a permanent, virus-agnostic Trm cellular safeguard in the lung parenchyma. Upon the emergence of a novel respiratory threat (“Day 0”), stockpiled, at-home AIR kits would be deployed immediately to primed patients. This patient-initiated mechanism acts as an instant, first-line mucosal barrier during the critical initial 100 days, mitigating healthcare surge capacity constraints while strain-specific technologies are still in development.

### 5.2. Evaluation of Autoimmune Risks and Localized Pulmonary Inflammation

A primary safety consideration regarding the administration of foreign allogeneic material is the potential induction of auto-reactive immune responses, persistent chimerism, or Graft-versus-Host Disease (GvHD). Crucially, because the intradermally injected AlloStim^®^ cells are completely mismatched to the host, they are rapidly recognized and cleared via an intact Host-versus-Graft (HvG) response, completely preventing long-term cell engraftment. Furthermore, the subsequent AIR phase utilizes a non-viable, fixed-antigen cell lysate rather than living cells; foreign HLA molecules are rapidly cleared by standard host macrophage phagocytosis within hours, preventing the chronic antigen exposure required to break peripheral tolerance.

However, because the platform systematically upregulates Th1 cell titers, a theoretical risk of exacerbating pre-existing Th1-mediated autoimmune diseases exists. To mitigate this, individuals with a documented medical history of autoimmune disease are strictly excluded from clinical enrollment. Reassuringly, to date, no de novo autoimmune adverse events have been reported in subjects without a baseline autoimmune history. Rather than fueling uncontrolled local tissue pathology, the rapid intracellular containment of early viral replication via the transient mucosal IFN-γ surge acts as an anti-inflammatory safety gatekeeper, preventing the massive, disorganized late-phase inflammatory cascade (“cytokine storm”) that typically drives severe clinical morbidity and ARDS in the senescent lung.

### 5.3. Proposed Phase II/III Randomized Trial Protocol Parameters

To validate this patient-initiated rescue model beyond a high-level concept, a randomized, double-blind, placebo-controlled Phase II/III clinical trial has been designed (ALLOCARE) with all-cause respiratory hospitalization as the primary clinical endpoint. The trial will specifically enroll elderly individuals (≥65 years old) residing in long-term care or congregate facilities. Notably, individuals with an official medical history (antecedents) of moderate-to-severe COPD or asthma will not be excluded; instead, they will be tracked and evaluated as a pre-specified, high-risk clinical subgroup to test efficacy in highly vulnerable phenotypes.

All participants will maintain their standard care regimens, including mandatory background vaccinations against seasonal influenza, pneumococcus (PCV21), and COVID-19. The intervention targets acute exacerbations driven by community-acquired seasonal viral pathogens (e.g., HRV, influenza, RSV) during peak winter transmission cycles. Patients randomized to the active arm will complete the 14-day intradermal Allopriming induction protocol prior to the winter season. Upon the earliest onset of any prodromal respiratory symptoms (defined as rhinorrhea, sore throat, or a ≥10% decline in baseline peak expiratory flow), patients will self-administer a single, reconstituted dose of AIR via an at-home vibrating mesh nebulizer.

### 5.4. Limitations and Challenges

Despite the robust immunological rationale detailed herein, several core limitations must be acknowledged. First, this platform represents a perspective concept based on systemic Phase I/II clinical trial parameters in healthy adults. There is currently a complete absence of direct human clinical data demonstrating the safety, tolerability, or pharmacokinetic distribution of aerosolized cellular lysates within diseased or hyper-reactive human lungs. Second, manufacturing scaling, cryogenic stability preservation, and strict batch-to-batch consistency across diverse healthy donor blood lots present significant operational and regulatory hurdles. Finally, ensuring precise patient compliance and proper nebulizer handling within a narrow, self-administered therapeutic window at home poses a real-world implementation challenge that requires rigorous validation in upcoming trials.

## 6. Conclusions

The global mortality burden of CRD is inextricably linked to the immunological vulnerabilities of an aging population. Current preventive strategies, while essential, remain constrained by the narrow therapeutic windows of strain-specific vaccines and the delayed innate responses of the senescent lung. By combining systemic Th1 remodeling with a targeted mucosal rescue, the Allopriming/AIR platform potentially bridges the “interferon gap” and establishes a self-amplifying shield of respiratory resilience. This virus-agnostic approach offers a scalable, at-home solution to protect the most vulnerable populations against the seasonal threats of today and the “Disease X” challenges of tomorrow.

## Figures and Tables

**Table 1:** Comparative Matrix of Preventive and Interventional Respiratory Strategies.

Evaluation Parameter	Traditional Seasonal Vaccines (e.g., *Influenza, RSV)*	Exogenous Interferon Therapy (e.g., *Inhaled* *IFN-α\β)*	Dual-Phase Cellular Adjuvant Platform *(Allopriming*/*AIR)*
Target Specificity	Pathogen-specific *(Relies on targeted antigen matching)*	Pathogen-agnostic *(Broad-spectrum antiviral activity)*	Pathogen-agnostic *(Broad-spectrum protective coverage)*
Therapeutic Window	Proactive induction *(Administered weeks/months prior to exposure)*	Exceptionally narrow post-exposure window *(Typically within hours of symptom onset)*	Immediate, patient-initiated *(Triggered at the earliest prodromal onset)*
Primary Immune Mechanism	Generation of neutralizing antibodies and localized systemic T-cell clones	Direct, passive chemical supplementation of transient antiviral mediators	Bystander activation and rapid mobilization of pre-positioned mucosal Th1 \(T_{rm}\) cells
Impact on Senescent Micro- environment	None *(Efficacy remains heavily compromised by age-related hyporesponsiveness)*	Transient and reactive *(Fails to correct baseline, long-term cellular dysregulation)*	Active cellular remodeling *(Shifts senescent Th2/inflammaging profile to a youthful Th1/DC1 rheostat)*
Vulnerability to Pathogen Mutation	High *(Prone to clinical failure driven by rapid antigenic drift/shift)*	Low *(Independent of surface protein variations)*	Negligible *(Pathogen-independent; addresses “Disease X” and unknown emerging variants)*
Risk of Localized Systemic Inflammation	Negligible *(Safe, targeted local response)*	Moderate-High *(Risk of uncontrolled* *hyper-cytokinemia or systemic rebound cascades)*	Low/Regulated *(Transient, self-limiting local IFN-γ surge acts as a kinetic gatekeeper)*

## Data Availability

No new data were created or analyzed in this study. The clinical data discussed in this manuscript are available upon reasonable request from the corresponding author.

## Abbreviations

The following abbreviations are used in this manuscript:

AIR	Alloantigen Inhalation Recall
ARDS	Acute Respiratory Distress Syndrome
BALF	Bronchoalveolar Lavage Fluid
cGMP	Current Good Manufacturing Practice
COPD	Chronic Obstructive Pulmonary Disease
CRD	Chronic Respiratory Disease
CRS	Cytokine Release Syndrome
DAMP	Damage-Associated Molecular Pattern
DC	Dendritic Cell
DC1	Type 1 Dendritic Cell
GOLD	Global Initiative for Chronic Obstructive Lung Disease
GvHD	Graft-versus-Host Disease
GvT	Graft-versus-Tumor
HRV	Human Rhinovirus
HSP	Heat Shock Protein
HvG	Host-versus-Graft
HvT	Host-versus-Tumor
iBALT	Inducible Bronchus-Associated Lymphoid Tissue
ICD	Immunogenic Cell Death
IFN	Interferon
ISR	Injection Site Reaction
LC	Langerhans Cell
LRT	Lower Respiratory Tract
MHC	Major Histocompatibility Complex
MMAD	Mass Median Aerodynamic Diameter
NK	Natural Killer cell
PAMP	Pathogen-Associated Molecular Pattern
PCV21	21-valent Pneumococcal Conjugate Vaccine
PRR	Pattern Recognition Receptor
ROS	Reactive Oxygen Species
RSV	Respiratory Syncytial Virus
SAE	Serious Adverse Event
SARS-CoV-2	Severe acute respiratory syndrome coronavirus 2
TCR	T-Cell Receptor
TGF	Transforming Growth Factor
Th1	T-helper 1
Th2	T-helper 2
Trm	Tissue-resident memory T cell
URT	Upper Respiratory Tract
VIE	Viral-Induced Exacerbation
